# Curcuminoids Activate TET Enzymes and Increase DNA Hydroxymethylation and Active Demethylation in Leukemia Cells

**DOI:** 10.3390/ijms27010310

**Published:** 2025-12-27

**Authors:** Sridhar A. Malkaram, Suhila Sawesi, Botao Peng, Badreldeen Rashrash, Hailey Cox, Tamer E. Fandy

**Affiliations:** 1 Department of Mathematics, Engineering & Computer Science, West Virginia State University, Institute, WV 25112, USA; smalkaram@wvstateu.edu; 2 Health Informatics and Bioinformatics Graduate Program, Department of Information Sciences and Technologies (IST), College of Computing, Grand Valley State University, Allendale, MI 49401, USA; sawesis@gvsu.edu; 3 School of Pharmacy, University of Charleston, Charleston, WV 25304, USA; 4 Paul L. Foster School of Medicine, Texas Tech University Health Sciences Center, El Paso, TX 79905, USA

**Keywords:** curcumin, dimethoxycurcumin, leukemia, DNA hydroxymethylation, active demethylation, TET enzymes, oxidative bisulfite sequencing (OxBS)

## Abstract

Curcuminoids demonstrate diverse pharmacological activity as antioxidant, neuroprotective, antitumor, and anti-inflammatory drugs. Dimethoxycurcumin (DMC) is a metabolically stable analog of curcumin, and both drugs modify the activity of several epigenetic enzymes that affect DNA methylation and histone modifications. 5-hydroxymethylcytosine (5hmC) is an epigenetic mark involved in active demethylation and in gene expression regulation. The effect of curcuminoids on the activity and expression of TET enzymes involved in 5hmC oxidation and active demethylation in leukemia cells is unclear. In this study, we investigated the impact of curcumin and DMC on the activity and expression of the three isoforms of TET enzymes. We also studied their effect on global 5hmC and performed a genome-wide analysis of 5hmC distribution at the single CpG level using oxidative bisulfite sequencing, which can differentiate between 5hmC and 5-methylcytosine. Both curcumin and DMC increased the activity and the mRNA expression of the three isoforms of TET. Concordantly, they also increased the global 5hmC level in leukemia cells. Single CpG analysis showed that both drugs induced a 5hmC increase and active demethylation at gene promoters, CpG islands and shores, exons, introns, and intergenic regions. Curcumin induced a promoter 5hmC increase in 194 genes and promoter-active demethylation in 154 genes. On the other hand, DMC induced a promoter 5hmC increase in 173 genes and promoter-active demethylation in 171 genes. Our study identifies curcuminoids as active demethylators through the activation of TET enzymes and provides a rationale for testing their combination with DNA hypomethylating agents in leukemia animal models.

## 1. Introduction

Curcuminoids represent a small class of plant secondary metabolites that includes curcumin, demethoxycurcumin, and bisdemethoxycurcumin, all isolated from the turmeric rhizome [1,2]. A growing body of evidence demonstrates that curcuminoids induce a variety of epigenetic modifications [3,4,5]. Curcuminoids demonstrate extensive biological activity as antioxidant, neuroprotective, antitumor, and anti-inflammatory agents [6,7,8,9]. Curcumin is the most widely used curcuminoid, and despite its proven efficacy and safety against numerous diseases, its poor bioavailability [10], rapid metabolism [11], and rapid systemic elimination limit its therapeutic efficacy. Therefore, several curcumin analogs were synthesized to improve its bioavailability and stability.

Dimethoxycurcumin (DMC) is a more metabolically stable and potent synthetic analog of curcumin [5,12,13]. DMC showed antitumor effects and induced epigenetic alterations in tumor cells [3,5,14]. The antitumor activity of both curcumin and DMC could be attributed to their abilities to reverse the epigenetic alterations associated with tumor pathogenesis. Several studies documented the epigenetic alterations induced by these compounds in leukemia cells, such as their impact on DNA methylation [5,14,15,16], histone acetylation [17,18], and miRNA modulation [19,20]. However, their effect on DNA hydroxymethylation is largely unknown.

DNA methylation was believed to be an irreversible epigenetic modification until the discovery of the ten-eleven translocation protein 1 (TET1) that catalyzes the successive oxidation of 5-methylcytosine (5mC) to 5-hydroxymethylcytosine (5hmC), 5-formylcytosine (5fC), and 5-carboxylcytosine (5caC) [21]. The TET protein family has three isoforms, namely, TET1, TET2, and TET3. These 5mC oxidation products serve as an intermediate in the conversion of 5mC to unmodified cytosines, a process known as active demethylation [22,23]. TET enzyme activity has emerged as an important tumor suppressor mechanism in cancer. TET2 is a frequently mutated gene in acute myeloid leukemia (AML) [24,25,26] and glioblastoma [27] and contributes to their pathogenesis. It is now recognized that all three TET isoforms are involved in a wide range of different cancer types. Accordingly, TET enzymes are rational targets for cancer therapy.

In this manuscript, we identified curcumin and DMC as active demethylators through the activation of TET enzymes. Both drugs increased the activity and induced the expression of the three TET isoforms in leukemia cells. Additionally, we detected a global increase in the 5hmC mark by both drugs. Genome-wide analysis of 5hmC at the single CpG level in leukemia cells revealed increases in 5hmC in CpG islands and promoters of leukemia-related genes. Moreover, decreases in 5hmC, indicating active demethylation, were similarly detected in promoters and CpG islands of leukemia-related genes. In conclusion, curcumin and DMC are capable of increasing DNA hydroxymethylation with consequent active demethylation of epigenetically silenced genes, which could contribute to their antitumor effect.

## 2. Results

### 2.1. Curcumin and DMC Increased Global DNA Hydroxymethylation in Leukemia Cells

The effect of curcumin and its analogs on DNA methylation reversal is controversial. Reversal of epigenetically silenced methylated genes was reported [28,29] but was not supported by later studies [5,15]. On the other hand, the effect of curcumin on DNA 5-hydroxymethylation (5hmC), an intermediate epigenetic mark that could also influence gene expression, is unclear [30]. Accordingly, we studied the effect of curcumin and its analog DMC on global DNA 5hmC in two leukemia cell lines to monitor any changes in this epigenetic mark.

**Figure 1:**
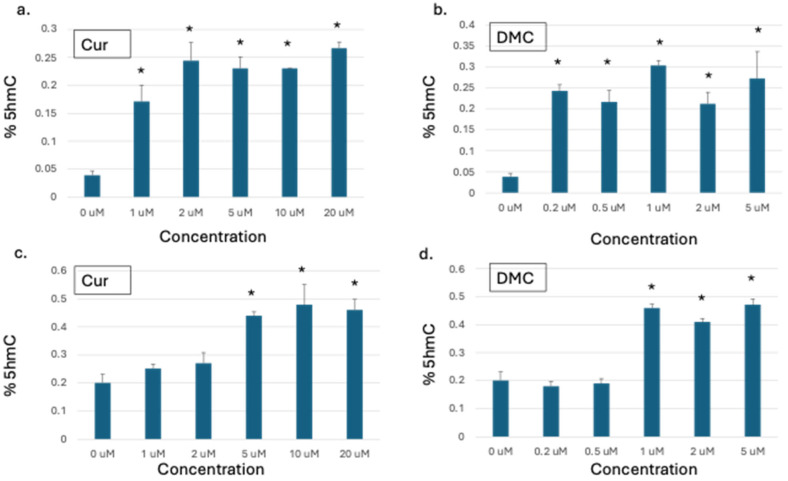
**Curcuminoids increase global 5hmC in leukemia cells.** Graded concentrations of curcumin (Cur) or DMC were used to treat U937 (**a**,**b**) and HL60 leukemia cells (**c**,**d**) for 48 h, followed by genomic DNA extraction and quantitation of global 5hmC as described in the methods. The data represent the mean of 3 replicates ± SD. * indicates a significant difference from the control at *p* < 0.05.

U937 and HL60 human leukemia cells were treated with graded concentrations of either curcumin or DMC for 48 h, followed by DNA extraction and quantitation of global DNA 5hmC, as described in the methods. The concentrations used for both drugs induced minimal apoptosis even at the highest concentration, as described previously [3]. U937 cells showed a significant increase in global DNA 5hmC at all the concentrations used for both curcumin and DMC (Figure 1a,b). However, the 5hmC increase was comparable at the different drug concentrations used and did not show a linear dose–response relationship. On the other hand, HL60 cells showed a similar increase in global DNA 5hmC but at higher concentrations compared to U937 (Figure 1c,d). Taken together, curcumin and DMC increased global DNA 5hmC in leukemia cells.

### 2.2. Curcumin and DMC Increased the Activity of TET Enzymes in Leukemia Cells

**Figure 2:**
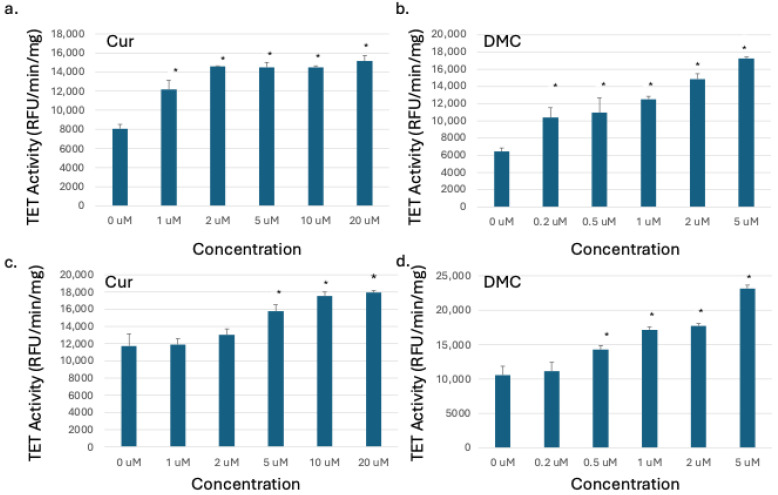
**Curcuminoids increase TET enzymatic activity in leukemia cells.** Graded concentrations of curcumin (Cur) or DMC were used to treat U937 (**a**,**b**) and HL60 leukemia cells (**c**,**d**) for 48 h, followed by nuclear protein extraction and quantitation of TET activity, as described in the methods. The data represent the mean of 3 replicates ± SD. * indicates significant difference from the control at *p* < 0.05.

The ten-eleven translocation (TET) enzyme family has three known isoforms (TET1, TET2, and TET3) involved in active DNA demethylation [31]. We detected a global 5hmC increase after curcumin or DMC treatment of leukemia cells, which is indirect evidence of TET enzyme activation. To confirm that, we measured the TET enzyme catalytic activity in the nuclear extracts of HL60 and U937 cells after 48 h treatment with graded concentrations of curcumin and DMC, as described in the methods. A significant increase in TET activity was observed by both drugs in U937 cells (Figure 2a,b). Even the lowest concentration of both drugs induced an increase in TET activity in U937. Similar to the global 5hmC results, HL60 cells were more resistant, and lower concentrations of curcumin or DMC did not induce TET activity (Figure 2c,d). Higher concentrations of both drugs induced an increase in TET activity, and the increase was concentration-dependent. In summary, an increase in TET activity was observed in the nuclear extracts of both cell lines after treatment with curcumin or DMC, which explains the previously observed increase in global DNA 5hmC.

### 2.3. Curcumin and DMC Induce TET Isoform Transcription in Leukemia Cells

Epigenetic modifiers, like 5-azacytidine and 5-aza-2′-deoxycytidine, induced the transcription of both the TET and DNMT enzyme isoforms in leukemia cells [5]. The observed increase in activity of TET enzymes by curcumin and DMC could be mediated through different mechanisms, like increasing the enzyme transcription, stabilization of mRNA of the enzyme, or direct binding of the drug to the enzyme. We hypothesized that curcumin and DMC induced the transcription of TET enzymes with a consequent increase in TET enzyme activity.

**Figure 3:**
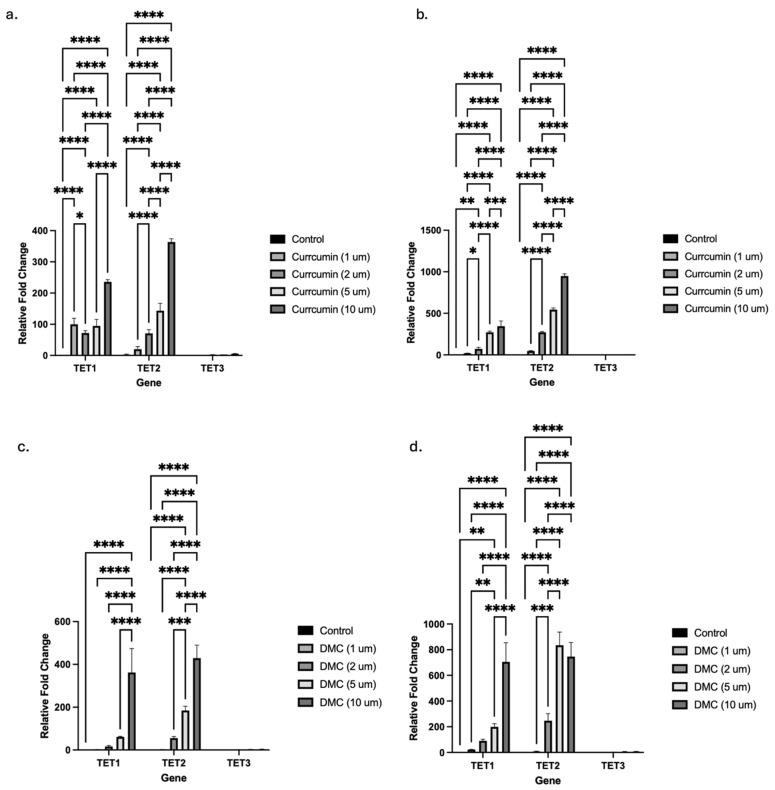
**Curcuminoids induce TET isoform transcription in leukemia cells.** U937 leukemia cells were treated with graded concentrations of either curcumin for 24 and 48 h (**a**) and (**b**), respectively) or DMC ((**c**) and (**d**), respectively), followed by RNA extraction and single-step RT-PCR, as described in the methods. The data represent the mean ± SD for 3 replicates. * indicates a significant difference at *p* < 0.05, ** indicates a significant difference at *p* < 0.01, *** indicates a significant difference at *p* < 0.001, and **** indicates a significant difference at *p* < 0.0001. Note that TET3 induction is not visible because of the scale.

To test our hypothesis, we performed qRT-PCR to quantify the effect of both drugs on the transcription of the three isoforms, TET1, TET2, and TET3, in U937 leukemia cells. Curcumin treatment for 24 or 48 h significantly induced mRNA expression of both the TET1 and TET2 isoforms at all the concentrations tested (Figure 3a and Figure 3b, respectively). The highest fold increase in mRNA expression was observed at the highest curcumin concentrations (5 and 10 µM), and the 48 h treatment induced higher fold induction compared to the 24 h treatment. TET3 isoform transcription was also induced, but with much lower fold induction compared to the other isoforms (the increase is not visible on the graphs because of the scale). Similar to the other TET isoforms, TET3 mRNA induction was also concentration-dependent, with the highest fold induction (6-fold) observed at the 10 µM concentration after 48 h of treatment (Supplementary Figure S1a).

Similarly, DMC induced the transcription of both the TET1 and TET2 isoforms to a greater extent compared to TET3 after 24 and 48 h of treatment (Figure 3c and Figure 3d, respectively). TET3 mRNA was increased by different DMC concentrations up to 3-fold after 24 h and 8-fold after 48 h (the increase is not visible on the graphs in Figure 3 because of the scale, and Supplementary Figure S1b shows the effect of DMC on TET3 mRNA induction). Collectively, both drugs significantly induced the transcription of the three TET isoforms; however, TET3 isoform mRNA induction was relatively much less than the other isoforms.

### 2.4. Genome-Wide Mapping of 5hmC at the Single CpG Level in Leukemia Cells Treated with Curcuminoids

The above data demonstrated an increase in global 5hmC in leukemia cells after treatment with curcumin or DMC. Unfortunately, the data measured only the overall changes in 5hmC without determining the genomic locations of the changes in 5hmC. Additionally, the method of analysis was not capable of detecting active demethylation. To overcome these drawbacks, we performed single CpG resolution mapping of 5hmC in U937 cells to monitor changes after treatment with curcumin (5 µM) or DMC (1 and 2 µM) for 48 h, as described in the methods. Data analysis was performed at three levels: single CpG dinucleotide analysis, CpG island (CpGi) analysis, and gene promoter analysis.

**Figure 4:**
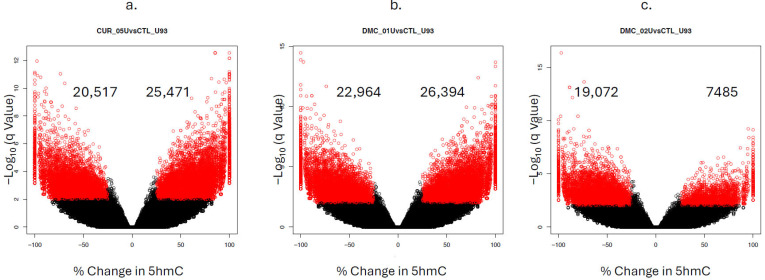
**Volcano plots for curcumin- and DMC-induced changes in 5hmC at the single-CpG level.** U937 cells were treated with curcumin (5 µM, (**a**)), DMC (1 µM, (**b**)), and DMC (2 µM, (**c**)) for 48 h, followed by oxidative bisulfite sequencing, as described in the methods. In the volcano plot, each black circle represents a CpG site with a nonsignificant change (*p* < 0.05) in 5hmC, while the red circles represent a CpG site with a significant change at *p* < 0.05. Positive values on the x-axis indicate an increase in 5hmC relative to the control untreated samples, and negative values indicate a decrease in 5hmC relative to the control (active demethylation). The numbers at the top indicate the number of CpG sites showing an increase in DNA 5hmC (positive values on the x-axis) or a decrease in DNA 5hmC (negative values on the x-axis).

For single CpG dinucleotide analysis, curcumin increased 5hmC at 25,471 CpG sites (positive values) and decreased 5hmC (negative values) at 20,517 CpG sites (indicating active demethylation) (Figure 4a). On the other hand, DMC (1 µM) similarly increased 5hmC at 26,394 CpG sites and decreased 5hmC at 22,964 CpG sites (Figure 4b). Increasing the concentration of DMC (2 µM) shifted the majority of 5hmC changes to negative values (19,072 vs. 7485), indicating an increase in active demethylation due to increased TET activity (Figure 4c). Furthermore, comparing the total number of CpG sites (both increases and decreases in 5hmC) between 1 µM and 2 µM DMC (Figure 4b,c) showed a decrease in the total number of CpG sites affected in the 2 µM concentration (26,557 versus 49,358 CpG sites). This difference is mainly attributed to the cytotoxicity of the higher concentration of DMC.

**Figure 5:**
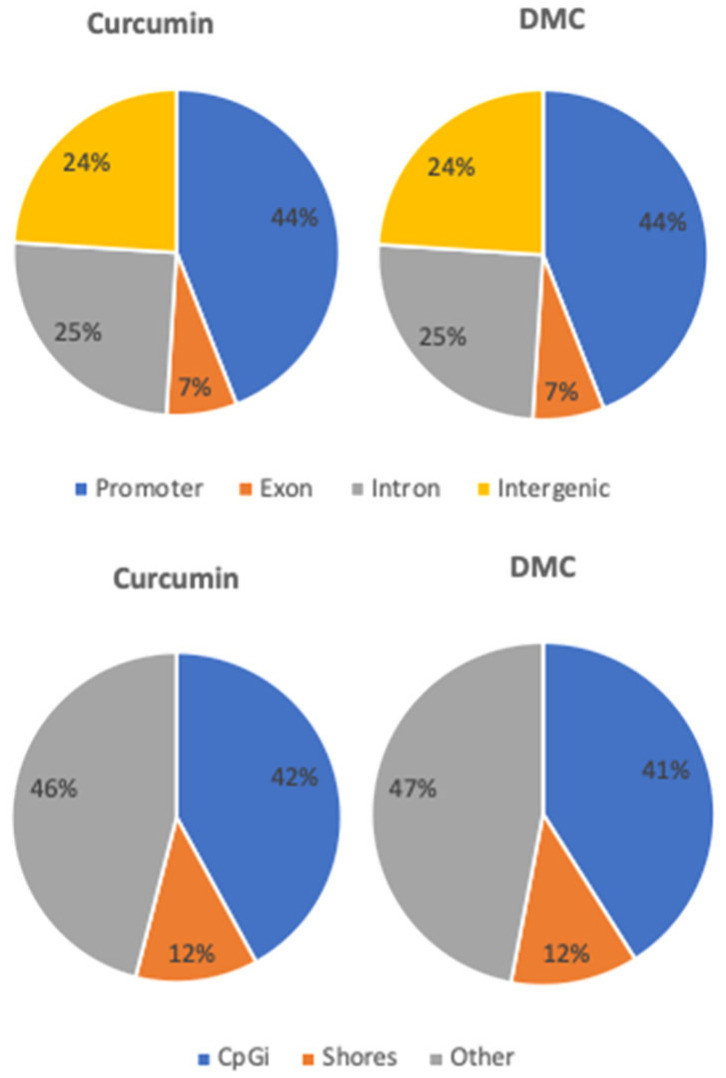
**Genomic distribution of the increase in 5hmC induced by curcumin and DMC in leukemia cells.** U937 leukemia cells treated by curcumin (Cur) (5 μM) or DMC (1 μM) for 48 h, followed by single CpG analysis of 5hmC distribution using RRBS, as described in the methods. The **upper** panel shows the distribution of the 5hmC increase in promoters, exons, introns, and intergenic regions after treatment with Cur or DMC. The **lower** panel shows the distribution of 5hmC increase in CpG islands (CpGi), shores, and other regions after treatment with Cur or DMC.

Both curcumin and DMC induced quite similar 5hmC increases at different genomic regions, where 44% and 7% of the increase in 5hmC happened at promoter regions and exons, respectively (Figure 5, upper panel). Similarly, 41–42% and 12% of the increase in 5hmC by both drugs occurred within CpG islands and shores, respectively (Figure 5, lower panel).

Active demethylation by both drugs, as represented by a decrease in 5hmC, was also observed in promoter regions and exons, where 41–42% of the total decrease in 5hmC occurred in promoter regions and 6–7% occurred in gene exons (Supplementary Figure S2). Moreover, 36–37% of active demethylation by both drugs occurred within CpG islands (Supplementary Figure S2).

**Figure 6:**
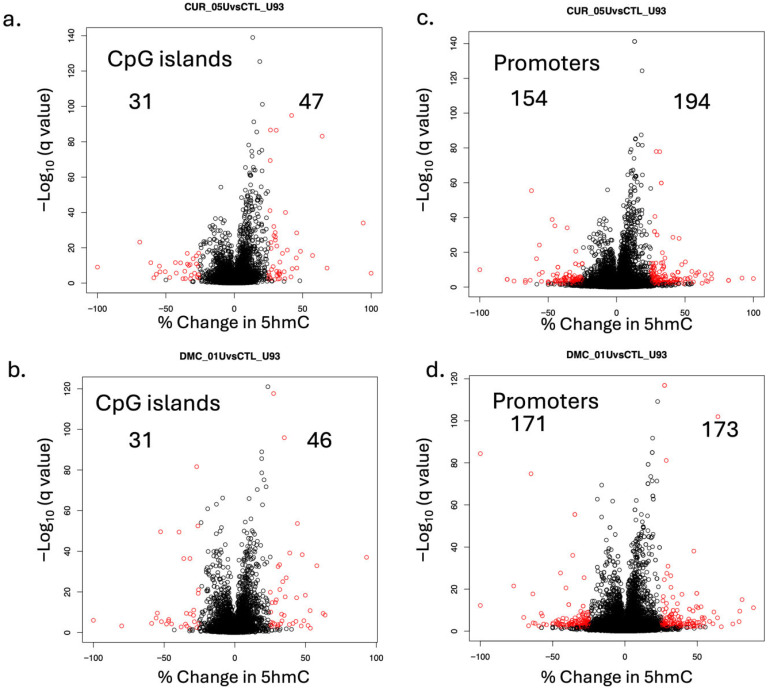
**Volcano plots for curcumin- and DMC-induced changes in 5hmC at CpG islands and promoter regions.** U937 cells were treated with curcumin (5 μM) and DMC (1 μM) for 48 h, followed by oxidative bisulfite sequencing, as described in the methods. For CpG island analysis, (**a**,**b**) represent curcumin and DMC treatment, respectively. For gene promoter analysis, (**c**,**d**) represent curcumin and DMC treatment, respectively. In the volcano plot, each black circle represents a CpG site with a nonsignificant change (*p* < 0.05) in 5hmC, while the red circles represent a CpG site with a significant change at *p* < 0.05. Positive values on the x-axis indicate an increase in 5hmC relative to the control untreated samples, and negative values indicate a decrease in 5hmC relative to the control (active demethylation). The numbers at the top indicate the number of CpG sites showing an increase in DNA 5hmC (positive values on the s-axis) or a decrease in DNA 5hmC (negative values on the x-axis).

Analysis of CpGi showed that curcumin increased 5hmC in 47 CpGis, while DMC increased 5hmC in 46 CpGis (Figure 6a and Figure 6b, respectively). Among these CpGis, four common CpGis showed an increase in 5hmC by both drugs. These four CpGis are located within the promoter region (≤1000 bp upstream TTS) of the following genes: *DKKL1*, *SLFN12L*, *IKZF4*, and *RNA5S1*. On the other hand, active demethylation was observed in 31 CpGis by both drugs (Figure 6a,b). Among these 31 CpGis, 17 common CpGis were actively demethylated by both drugs. Overall, 5 of these 17 CpGis were located in the promoter region of the following genes: *LINC02610*, *ZNF222*, *WASIR1*, *GCFC2*, and *RNVU1-14*.

Promoter region analysis of 5hmC in leukemia cells treated with curcumin or DMC showed significant increases and decreases in 5hmC in several gene promoters. Curcumin showed an increase in promoter 5hmC in 194 genes and a decrease (active demethylation) in 154 genes (Figure 6c). Similarly, DMC showed an increase in promoter 5hmC in 173 genes and a decrease in 171 gene promoters (Figure 6d). Among these genes, 34 and 29 leukemia-related genes showed promoter active demethylation by curcumin (Table 1) and DMC (Table 2), respectively. Supplementary Tables S1 and S2 show the list of leukemia-related genes with promoter 5hmC increase after DMC and curcumin treatment, respectively.

### 2.5. KEGG Pathway Enrichment Analysis

The KEGG database was used to map the set of genes that showed significant changes in 5hmC to signaling pathways. Both curcumin and DMC modulated 5hmC in a set of genes that was mapped to common signaling pathways related to AML and leukemia, like Wnt signaling, Hippo signaling, Rap1 signaling, cAMP signaling, and signaling pathways regulating pluripotency of stem cells (Supplementary Figure S3).

## 3. Discussion

In this manuscript, we monitored the effect of curcumin and its metabolically stable analog DMC on the enzymatic activity and transcription of different TET isoforms and how the increased TET activity impacted 5hmC changes genome-wide at the single CpG level. We used oxidative bisulfite sequencing to differentiate between 5mC and 5hmC. Both drugs increased the conversion of 5mC into 5hmC and also the active demethylation of 5hmC into cytosine. Increasing the concentration of DMC induced a significant increase in active demethylation compared to the 5hmC increase, which is consistent with the observed dose-dependent increase in TET activity by DMC. Both drugs induced active demethylation and increased 5hmC at the promoters of several leukemia-related genes. These 5hmC changes could reverse or suppress epigenetic silencing induced by DNA methylation and contribute to the antitumor effect of curcuminoids.

Initially, 5hmC was considered a passive intermediate in the process of active demethylation, but later, it was recognized as a bona fide dynamic epigenetic mark that affects gene expression, hematopoiesis, and stem cell pluripotency [32,33]. In contrast to 5mC, 5hmC is more enriched in euchromatin, with active histone marks (like H3K4me3 and H3K27ac) and actively transcribed genes. Oxidation of 5mC into 5hmC inhibits the binding of methyl-CpG-binding proteins (MBD1, MBD2, and MBD4) and the recruitment of gene repression complexes. Indeed, 5hmC is often detected in promoters and enhancers of actively transcribed genes, and its presence is associated with increased gene expression. Moreover, specific proteins like MBD3 and UHRF2 act as epigenetic readers for 5hmC and contribute to transcriptional activation [34]. Loss of UHRF2 expression has been implicated in neoplasia [35], where alterations in its ability to recognize and interpret 5hmC may contribute to promoter hypermethylation, disrupted gene expression, and tumor progression. In this study, we detected significant increases in 5hmC and active DNA demethylation after treatment with curcuminoids at promoters, CpGis, exons, introns, and intergenic regions. These 5hmC changes could be valuable in leukemia subtypes characterized by promoter hypermethylation by restoring the expression of genes silenced by DNA methylation. Nonetheless, a drawback of the observed 5hmC increase and active demethylation is the possible unintended enhanced expression of oncogenes, which is undesirable. For instance, genes like *ANGPT1*, *PES1*, and *ZNF222* demonstrated promoter active demethylation by both compounds and are known for promoting cell survival and proliferation of different types of leukemia [36,37,38]. This drawback was also observed with other classes of anticancer drugs, like DNA hypomethylating agents and HDAC inhibitors, where targeted locus-specific delivery is not feasible. Predicting the net effect of the mixed active demethylation of tumor suppressor genes and oncogenes on tumor progression requires in vivo animal and clinical testing.

TET enzymes are essential for the oxidation of 5mC into 5hmC and the sequential oxidative steps of active demethylation [22]. Loss-of-function mutations in TET2 were frequently observed in myeloid malignancies and resulted in decreased 5hmC levels, increased 5mC levels, and impaired hematopoietic differentiation [39]. Our study suggests that the use of curcuminoids in leukemia cells with wild-type or partially functioning TET2 could increase 5hmC levels and induce active demethylation of silenced promoter hypermethylated genes. Similarly, IDH1 and IDH2 gain-of-function mutations in leukemia lead to the accumulation of the competitive inhibitor of TET enzymes, 2-hydroxyglutarate (2-HG), with consequent DNA hypermethylation. The observed increase in the transcription and the activity of TET enzymes by curcuminoids could overcome the competitive inhibition by the oncometabolite 2-HG. There are several mechanisms that could contribute to the observed enhanced TET transcription. Increased transcription factor binding, histone modifications, chromatin remodeling, enhancer activation, and promoter–enhancer looping are possible mechanisms that could contribute to enhanced TET transcription. We previously reported that both curcumin and DMC inhibited the methylation of the transcriptional repressor marks H3K9 and H3K27 [3]. Direct promoter/transcription factor binding is also a possible mechanism, but it would be problematic to investigate due to the promiscuous non-specific binding of curcumin [40].

Previous studies demonstrated a relationship between chemotherapy resistance and 5hmC levels. Chemoresistance in hepatocellular carcinoma was associated with reduced 5hmC levels [41]. Moreover, AML patients treated with 5-azacytidine in combination with standard chemotherapy showed improved outcomes in patients with favorable 5hmC patterns [42]. Accordingly, the combination of curcuminoids with chemotherapy could overcome chemoresistance associated with reduced 5hmC levels through activation of TET enzymes and increasing 5hmC levels.

Our results indicate that the effect of curcumin and DMC on 5hmC distribution was not identical but shared several similarities. These differences were expected due to differences in dosing, metabolic stability, and TET activation. In support of that, increasing the concentration of DMC was also associated with changes in 5hmC distribution compared to the lower concentration. Nevertheless, comparing the overall changes in 5hmC (active demethylation and increase in 5hmC) at promoters, CpGis, shores, introns, and exons showed quite similar changes by both drugs. To the best of our knowledge, there are no previous reports on the effect of DMC on 5hmC to compare with our findings. However, a few studies reported the effect of curcumin on 5hmC in other tissues. A previous study demonstrated a similar effect of curcumin on 5hmC in cultured vascular smooth muscle cells (VSMCs), where curcumin treatment blocked the effect of the CCDC80-induced reduction in TET2 and 5hmC by restoring the expression of TET2 in VSMCs [43]. Moreover, curcumin was shown to upregulate the expression of both the TET2 and TET3 enzymes in gastric cancer cell lines, with consequent active demethylation and re-expression of the tumor suppressor RB1 [44].

DMC differs from curcumin in chemical structure by replacing the two phenolic hydroxyl groups in curcumin with two methoxy groups. Accordingly, DMC is less vulnerable to phase-II conjugation and oxidative degradation [5]. The higher metabolic stability of DMC is concordant with the comparable 5hmC changes induced by both curcumin and DMC, using a DMC concentration that is five times less than that of curcumin. Moreover, DMC induced significantly higher apoptosis induction in leukemia cells compared to curcumin at equimolar concentrations [5]. Across other types of cancer, DMC showed higher potency compared to curcumin at an equimolar dose. For instance, in breast cancer cells, DMC induced paraptosis-like cell death and induced more potent inhibition of the proteasome compared to curcumin [45]. The epigenetic changes induced by both compounds shared some similarities but were not identical. Although both compounds increased the activity of TET enzymes, global 5hmC, and active demethylation, the affected genes were different, with minor overlapping. Similarly, mass spectrometry analysis of a variety of histone post-translational modifications induced by both drugs showed major differences despite similar inhibition of histone lysine methyltransferase (HKMT) and activation of different histone lysine demethylases, like JARID and LSD1 [3].

In this study, we used two different approaches—the global and locus-specific approaches—to study the impact of curcumin and DMC on 5hmC. The global approach was used to compare the global 5hmC changes across different concentrations of both compounds and leukemia cell lines. Despite its affordability and ability to detect 5hmC changes across different concentrations, it did not capture locus-specific 5hmC changes and just provided the net change in 5hmC without discriminating between 5hmC-decreased and 5hmC-increased loci. Accordingly, 5hmC mapping at the single CpG level using oxidative bisulfite sequencing was used to overcome this drawback.

CpGi analysis of 5hmC revealed both increases in 5hmC and active demethylation in the promoter region of several leukemia-related genes after curcuminoid treatment. For instance, the *BEX1* promoter showed an increase in 5hmC and was reported to act as a novel suppressor of oncogenic FLT3-ITD-driven AML [46]. Similarly, the Wnt signaling pathway inhibitor *sFRP4* showed an increase in 5hmC and was reported to be promoter-methylated in chronic myeloid leukemia. *PRKX* also showed an increase in 5hmC and was shown to play an essential role in myeloid cell differentiation, which is usually blocked or impaired in AML [47]. Other genes like *ZNF292*, *PRDM1*, and *CDH11* demonstrated promoter active demethylation and were previously linked to different leukemia types, where *PRDM1* and *CDH11* were reported to be frequently epigenetically silenced in these tumors [48,49].

In conclusion, curcuminoids activated and increased the expression of all the TET isoforms in leukemia cells, with a consequent increase in 5hmC and active demethylation of several leukemia-related genes and genes mapped to common signaling pathways related to leukemia. Our findings provide a rationale for future preclinical testing of the combination of curcuminoids with DNA hypomethylating agents to harness their combined effect on active demethylation and methylation reversal of hypermethylated genes.

## 4. Materials and Methods

### 4.1. Chemicals

Curcumin analytical standard grade (Sigma-Aldrich, Milwaukee, WI, USA) and DMC (Cayman, Ann Arbor, MI, USA) were both dissolved in DMSO as a 10 mM stock solution and then aliquoted and stored in the dark at −80 °C.

### 4.2. Cell Culture and Treatments

Human promyeloid leukemia (HL60) and human monocytic leukemia (U937) were obtained from the American Type Culture Collection (ATCC, Manassas, VA, USA) and cultured in RPMI-1640 medium (Sigma-Aldrich, Milwaukee, WI, USA) containing 10% fetal bovine serum (FBS) and 2.5 mM L-glutamine. The cells were maintained in a humidified incubator with 5% CO_2_ at 37 °C. The cells were treated with different micromolar concentrations of DMC, curcumin, or DMSO (control), followed by nuclear protein extractions to quantify global 5hmC or measure TET enzymatic activity, as described below.

### 4.3. Nuclear Protein Extraction

Nuclear extracts from the treated and untreated HL60 and U937 leukemia cells were prepared using an EpiQuikTM nuclear extraction kit (Epigentek, Farmingdale, NY, USA) as per the manufacturer’s protocol. Approximately 2 × 10^6^ cells were collected and centrifuged at 1000 rpm for 5 min. The nuclear extract was prepared and stored at −80 °C until use. The protein concentration of the nuclear extract was quantitated using a Quick Start Bradford Protein Assay (BioRad, Hercules, CA, USA).

### 4.4. Global DNA Hydroxymethylation Quantification

Fluorometric detection of global DNA 5-hydroxymethylation of cytosine (5hmC) was performed using a commercially available kit (Epigentek, NY, USA). Briefly, genomic DNA was extracted from HL60 and U937 cells treated with curcumin, DMC, or DMSO (control) and incubated in microplate wells treated to have high affinity for hydroxymethylated DNA but not methylated or unmethylated DNA. The bound 5hmC was quantified using a capture antibody followed by a detection antibody that was fluorescence-labeled.

### 4.5. TET Activity Quantification Assay

A fluorometric assay (Abcam, Waltham, MA, USA) was used to detect the activity of the TET isoforms using nuclear extracts from HL60 and U937 leukemia cells. Briefly, microplate wells coated with a methylated substrate were incubated with nuclear extracts from the leukemia cells treated with graded concentrations of curcumin, DMC, or DMSO (control) for 48 h. The methylated substrate was converted into a hydroxymethylated substrate, which is proportional to the enzymatic activity and can be quantified by incubation with a specific capture antibody followed by a fluorescent-labeled detection antibody. The activity for the TET enzymes was determined using the following formula:

$$TETActivity(RFU/min/mg)=\frac{\left(SampleRFU-blankRFU\right)}{\left(proteinamount(\mu g)\times min*\right)}\times 1000$$

where * Incubation time = 120 min and RFU = Relative Fluorescence Unit.

### 4.6. Real-Time Quantitative RT-PCR

Total RNA was extracted from the treated and control cells using RNeasy columns (Qiagen, Chatsworth, CA, USA). One-step qRT-PCR was performed as recommended by the manufacturer of a commercially available kit (ThermoFisher, Waltham, MA, USA) using a QuantStudio 5 Real-Time thermal cycler. The following primers were used for TET1: forward 5′cacaccagctccactgaaga3′ and reverse 5′ctccatcacaggagcagaca3′; for TET2: forward 5′ttggacttctgtgctcatgc3′ and reverse 5′ctcctgagcttccacactcc3′; and for TET3: forward 5′tgtgacgttgtcgagagagg3′ and reverse 5′attcccctctgtgtgtcctg3′. GAPDH was used as a loading control.

### 4.7. Single CpG Resolution Analysis of 5hmC Using Oxidative Bisulfite Sequencing

The ovation reduced representation bisulfite sequencing (RRBS) Methyl-Seq System (Nugen, Tecan Genomics, Redwood City, CA, USA) was used. RRBS is a technique that reduces the amount of sequencing compared to whole-genome sequencing and utilizes the methylation-insensitive restriction enzyme MspI, which recognizes CCGG [50]. Regular bisulfite sequencing is not capable of differentiating 5-methylcytosine (5mC) from 5hmC because cytosine will not be converted into thymine in both cases. In order to differentiate between 5mC and 5hmC, each sample was divided into two equal portions. The first portion (mock sample) underwent regular bisulfite treatment, followed by next-generation sequencing (provides a total of 5mC and 5hmC). The second portion (oxidative sample) underwent an oxidation step according to the manufacturer’s instructions to convert 5hmC into 5fC, followed by bisulfite treatment and next-generation sequencing. The conversion of 5hmC into 5fC allows for the conversion of cytosine into thymine after bisulfite treatment, and, consequently, comparing the cytosines in the first and second portions differentiates the 5hmC mark from the 5mC.

Paired-end bisulfite sequencing reads were pre-processed with FastQC v0.11.9, cutadapt v2.10, and Trim Galore v0.6.10 python functions to remove adapter content and simultaneously trim low-quality ends and filter for reads below Q20. The additional diversity adaptors were trimmed using a NuMetRRBS python script using recommended settings for paired-end reads [51]. The hg38 version of the human genome was used to align the reads. Before the alignment, the genome was converted to bisulfite-compatible versions using Bismark v0.22.3. The reads were aligned to the genome sequences using bowtie2 v2.4.4 via the Bismark scripts using default parameters. Methylation calling and extraction of methylation levels were performed using a Bismark methylation extractor. The methylation data (CpG sites) from all samples were merged to find the common sites present in all samples. After filtering for sites with <2 standard deviations and known C2T mutation sites, there were about 288,097 CpG sites left for further analysis. Analysis of CpG methylation with respect to genomic features was performed using the methylKit v1.34.0 and methylGSA v1.20.0 R packages.

Initially, the mock and oxidative samples were merged to obtain the common CpG sites. The difference in the methylation level at CpG sites between the mock and oxidative samples was taken as the hydroxymethylation level. The differential hydroxymethylation levels between the control and treatments were computed using the MethylKit v1.34.0 R package. Differential analysis was performed at 4 levels: base pairs, CpG islands, promoters, and 1000 bp tiles. The R package methylGSA v1.20.0, was used to compute Gene Set Enrichment (GSA) and pathway enrichment for the significantly different sites.

### 4.8. Statistical Analyses

All cell culture experiments were performed in triplicate. Data represent the mean ± standard deviation (S.D.). Multiple comparisons between the effects of different concentrations of curcumin or DMC on the mRNA expression of TET proteins were performed using one-way ANOVA followed by post hoc analysis using the Bonferroni test.

## Figures and Tables

**Table 1:** **Promoter active demethylation of leukemia-related genes by curcumin.** U937 leukemia cells treated with curcumin (5 µM) for 48 h, followed by genome-wide analysis of 5hmC, as described in the methods. Only genes with a *q*-value less than or equal to 0.05 were considered significantly different from the control. Yellow highlights indicate common genes affected by both curcumin and DMC.

Gene Name	Symbol	*q*-Value
alanine- and arginine-rich domain containing protein	AARD	0.0005
aldo-keto reductase family 1 member C1	AKR1C1	0.0000
angiopoietin 1	ANGPT1	0.0000
coiled-coil domain containing 89	CCDC89	0.0045
glycoprotein hormones, alpha polypeptide	CGA	0.0000
C-type lectin domain containing 10A	CLEC10A	0.0459
cannabinoid receptor 1	CNR1	0.0000
cysteine- and glycine-rich protein 1	CSRP1	0.0001
cystatin D	CST5	0.0121
major histocompatibility complex, class II, DR beta 5	HLA-DRB5	0.0000
kelch-like family member 4	KLHL4	0.0156
kallikrein related peptidase 14	KLK14	0.0032
keratin-associated protein 9-9	KRTAP9-9	0.0000
long intergenic non-protein coding RNA 1533	LINC01533	0.0002
long intergenic non-protein coding RNA 2714	LINC02714	0.0001
long intergenic non-protein coding RNA 2864	LINC02864	0.0007
MEF2C antisense RNA 1	MEF2C-AS1	0.0000
microRNA 1302-8	MIR1302-8	0.0305
microRNA 130a	MIR130A	0.0164
microRNA 138-1	MIR138-1	0.0001
membrane spanning 4-domains A3	MS4A3	0.0000
NPL4 homolog, ubiquitin recognition factor	NPLOC4	0.0014
olfactory receptor family 2 subfamily H member 1	OR2H1	0.0000
phosphate cytidylyltransferase 1B, choline	PCYT1B	0.0000
pescadillo ribosomal biogenesis factor 1	PES1	0.0000
phosphatidylserine decarboxylase	PISD	0.0004
PPP2R2B intronic transcript 1	PPP2R2B-IT1	0.0055
pregnancy-specific beta-1-glycoprotein 5	PSG5	0.0000
retinaldehyde-binding protein 1	RLBP1	0.0061
solute carrier organic anion transporter family member 1B3	SLCO1B3	0.0029
tumor protein D52	TPD52	0.0000
tripartite motif family like 1	TRIML1	0.0005
ubiquitin-specific peptidase 6	USP6	0.0003
zinc finger protein 222	ZNF222	0.0000

**Table 2:** **Promoter active demethylation of leukemia-related genes by DMC.** U937 leukemia cells treated with DMC (1 µM) for 48 h, followed by genome-wide analysis of 5hmC, as described in the methods. Only genes with a *q*-value less than or equal to 0.05 were considered significantly different from the control. Yellow highlights indicate common genes affected by both curcumin and DMC.

Gene Name	Symbol	*q*-Value
aldo-keto reductase family 1 member C1	AKR1C1	0.0005
angiopoietin 1	ANGPT1	0.0000
calcium/calmodulin-dependent protein kinase II beta	CAMK2B	0.0011
coiled-coil domain containing 89	CCDC89	0.0012
cyclin B3	CCNB3	0.0000
C-type lectin domain containing 10A	CLEC10A	0.0208
colipase	CLPS	0.0000
cannabinoid receptor 1	CNR1	0.0003
cysteine- and glycine-rich protein 1	CSRP1	0.0000
defensin alpha 1	DEFA1	0.0017
epidermal growth factor	EGF	0.0198
FLVCR choline and putative heme transporter 2	FLVCR2	0.0106
major histocompatibility complex, class II, DR beta 5	HLA-DRB5	0.0000
keratin 80	KRT80	0.0000
long intergenic non-protein coding RNA 570	LINC00570	0.0355
long intergenic non-protein coding RNA 2610	LINC02610	0.0000
long intergenic non-protein coding RNA 2864	LINC02864	0.0000
uncharacterized LOC286359	LOC286359	0.0015
membrane spanning 4-domains A3	MS4A3	0.0000
microtubule-associated scaffold protein 1	MTUS1	0.0000
olfactory receptor family 10 subfamily X member 1	OR10X1	0.0002
protein disulfide isomerase family A member 4	PDIA4	0.0317
pescadillo ribosomal biogenesis factor 1	PES1	0.0000
PPP2R2B intronic transcript 1	PPP2R2B-IT1	0.0077
small nucleolar RNA, C/D box 115-2	SNORD115-2	0.0002
tripartite motif family like 1	TRIML1	0.0000
ubiquitin-specific peptidase 17-like family member 2	USP17L2	0.0095
UDP-glucuronate decarboxylase 1	UXS1	0.0042
zinc finger protein 222	ZNF222	0.0000

## Data Availability

The original contributions presented in this study are included in this article/the Supplementary Materials. Further inquiries can be directed to the corresponding author.

## Supplementary Materials

The following supporting information can be downloaded at https://www.mdpi.com/article/10.3390/ijms27010310/s1.

## References

[1] Rao C.V., Rivenson A., Simi B., Reddy B.S. (1995). Chemoprevention of colon carcinogenesis by dietary curcumin, a naturally occurring plant phenolic compound. Cancer Res..

[2] Sudarshan K., Yarlagadda S., Sengupta S. (2024). Recent Advances in the Synthesis of Diarylheptanoids. Chem. Asian J..

[3] Sawesi S., Malkaram S.A., Abd Elmageed Z.Y., Fandy T.E. (2022). Modulation of the activity of histone lysine methyltransferases and demethylases by curcumin analog in leukaemia cells. J. Cell Mol. Med..

[4] Uddin M.G., Fandy T.E. (2021). DNA methylation inhibitors: Retrospective and perspective view. Adv. Cancer Res..

[5] Hassan H.E., Carlson S., Abdallah I., Buttolph T., Glass K.C., Fandy T.E. (2015). Curcumin and dimethoxycurcumin induced epigenetic changes in leukemia cells. Pharm. Res..

[6] Amalraj A., Pius A., Gopi S., Gopi S. (2017). Biological activities of curcuminoids, other biomolecules from turmeric and their derivatives—A review. J. Tradit. Complement. Med..

[7] Jakubczyk K., Druzga A., Katarzyna J., Skonieczna-Zydecka K. (2020). Antioxidant Potential of Curcumin-A Meta-Analysis of Randomized Clinical Trials. Antioxidants.

[8] Peng Y., Ao M., Dong B., Jiang Y., Yu L., Chen Z., Hu C., Xu R. (2021). Anti-Inflammatory Effects of Curcumin in the Inflammatory Diseases: Status, Limitations and Countermeasures. Drug Des. Dev. Ther..

[9] Sudarshan K., Perumal G., Aidhen I.S., Doble M. (2018). Synthesis of Unsymmetrical Linear Diarylheptanoids and their Enantiomers and Antiproliferative Activity Studies. Eur. J. Org. Chem..

[10] Dei Cas M., Ghidoni R. (2019). Dietary Curcumin: Correlation between Bioavailability and Health Potential. Nutrients.

[11] Fanca-Berthon P., Tenon M., Bouter-Banon S.L., Manfre A., Maudet C., Dion A., Chevallier H., Laval J., van Breemen R.B. (2021). Pharmacokinetics of a Single Dose of Turmeric Curcuminoids Depends on Formulation: Results of a Human Crossover Study. J. Nutr..

[12] Tamvakopoulos C., Dimas K., Sofianos Z.D., Hatziantoniou S., Han Z., Liu Z.L., Wyche J.H., Pantazis P. (2007). Metabolism and anticancer activity of the curcumin analogue, dimethoxycurcumin. Clin. Cancer Res..

[13] Pae H.O., Jeong S.O., Kim H.S., Kim S.H., Song Y.S., Kim S.K., Chai K.Y., Chung H.T. (2008). Dimethoxycurcumin, a synthetic curcumin analogue with higher metabolic stability, inhibits NO production, inducible NO synthase expression and NF-kappaB activation in RAW264.7 macrophages activated with LPS. Mol. Nutr. Food Res..

[14] Hassan H.E., Keita J.A., Narayan L., Brady S.M., Frederick R., Carlson S., Glass K.C., Natesan S., Buttolph T., Fandy T.E. (2016). The combination of dimethoxycurcumin with DNA methylation inhibitor enhances gene re-expression of promoter-methylated genes and antagonizes their cytotoxic effect. Epigenetics.

[15] Link A., Balaguer F., Shen Y., Lozano J.J., Leung H.C., Boland C.R., Goel A. (2013). Curcumin modulates DNA methylation in colorectal cancer cells. PLoS ONE.

[16] Khor T.O., Huang Y., Wu T.Y., Shu L., Lee J., Kong A.N. (2011). Pharmacodynamics of curcumin as DNA hypomethylation agent in restoring the expression of Nrf2 via promoter CpGs demethylation. Biochem. Pharmacol..

[17] Balasubramanyam K., Varier R.A., Altaf M., Swaminathan V., Siddappa N.B., Ranga U., Kundu T.K. (2004). Curcumin, a novel p300/CREB-binding protein-specific inhibitor of acetyltransferase, represses the acetylation of histone/nonhistone proteins and histone acetyltransferase-dependent chromatin transcription. J. Biol. Chem..

[18] Reuter S., Gupta S.C., Park B., Goel A., Aggarwal B.B. (2011). Epigenetic changes induced by curcumin and other natural compounds. Genes Nutr..

[19] Sun M., Estrov Z., Ji Y., Coombes K.R., Harris D.H., Kurzrock R. (2008). Curcumin (diferuloylmethane) alters the expression profiles of microRNAs in human pancreatic cancer cells. Mol. Cancer Ther..

[20] Zhang J., Zhang T., Ti X., Shi J., Wu C., Ren X., Yin H. (2010). Curcumin promotes apoptosis in A549/DDP multidrug-resistant human lung adenocarcinoma cells through an miRNA signaling pathway. Biochem. Biophys. Res. Commun..

[21] Tahiliani M., Koh K.P., Shen Y., Pastor W.A., Bandukwala H., Brudno Y., Agarwal S., Iyer L.M., Liu D.R., Aravind L. (2009). Conversion of 5-methylcytosine to 5-hydroxymethylcytosine in mammalian DNA by MLL partner TET1. Science.

[22] He Y.F., Li B.Z., Li Z., Liu P., Wang Y., Tang Q., Ding J., Jia Y., Chen Z., Li L. (2011). Tet-mediated formation of 5-carboxylcytosine and its excision by TDG in mammalian DNA. Science.

[23] Ito S., Shen L., Dai Q., Wu S.C., Collins L.B., Swenberg J.A., He C., Zhang Y. (2011). Tet proteins can convert 5-methylcytosine to 5-formylcytosine and 5-carboxylcytosine. Science.

[24] Ko M., Huang Y., Jankowska A.M., Pape U.J., Tahiliani M., Bandukwala H.S., An J., Lamperti E.D., Koh K.P., Ganetzky R. (2010). Impaired hydroxylation of 5-methylcytosine in myeloid cancers with mutant TET2. Nature.

[25] Ko M., An J., Pastor W.A., Koralov S.B., Rajewsky K., Rao A. (2015). TET proteins and 5-methylcytosine oxidation in hematological cancers. Immunol. Rev..

[26] Weissmann S., Alpermann T., Grossmann V., Kowarsch A., Nadarajah N., Eder C., Dicker F., Fasan A., Haferlach C., Haferlach T. (2012). Landscape of TET2 mutations in acute myeloid leukemia. Leukemia.

[27] Garcia M.G., Carella A., Urdinguio R.G., Bayon G.F., Lopez V., Tejedor J.R., Sierra M.I., Garcia-Torano E., Santamarina P., Perez R.F. (2018). Epigenetic dysregulation of TET2 in human glioblastoma. Oncotarget.

[28] Liu Z., Xie Z., Jones W., Pavlovicz R.E., Liu S., Yu J., Li P.K., Lin J., Fuchs J.R., Marcucci G. (2009). Curcumin is a potent DNA hypomethylation agent. Bioorg Med. Chem. Lett..

[29] Jha A.K., Nikbakht M., Parashar G., Shrivastava A., Capalash N., Kaur J. (2010). Reversal of hypermethylation and reactivation of the RARbeta2 gene by natural compounds in cervical cancer cell lines. Folia Biol..

[30] Hahn M.A., Qiu R., Wu X., Li A.X., Zhang H., Wang J., Jui J., Jin S.G., Jiang Y., Pfeifer G.P. (2013). Dynamics of 5-hydroxymethylcytosine and chromatin marks in Mammalian neurogenesis. Cell Rep..

[31] Gao Q., Shen K., Xiao M. (2024). TET2 mutation in acute myeloid leukemia: Biology, clinical significance, and therapeutic insights. Clin. Epigenetics.

[32] Shen L., Zhang Y. (2013). 5-Hydroxymethylcytosine: Generation, fate, and genomic distribution. Curr. Opin. Cell Biol..

[33] Nakauchi Y., Azizi A., Thomas D., Corces M.R., Reinisch A., Sharma R., Cruz Hernandez D., Kohnke T., Karigane D., Fan A. (2022). The Cell Type-Specific 5hmC Landscape and Dynamics of Healthy Human Hematopoiesis and TET2-Mutant Preleukemia. Blood Cancer Discov..

[34] Chen R., Zhang Q., Duan X., York P., Chen G.D., Yin P., Zhu H., Xu M., Chen P., Wu Q. (2017). The 5-Hydroxymethylcytosine (5hmC) Reader UHRF2 Is Required for Normal Levels of 5hmC in Mouse Adult Brain and Spatial Learning and Memory. J. Biol. Chem..

[35] Lu H., Bhoopatiraju S., Wang H., Schmitz N.P., Wang X., Freeman M.J., Forster C.L., Verneris M.R., Linden M.A., Hallstrom T.C. (2016). Loss of UHRF2 expression is associated with human neoplasia, promoter hypermethylation, decreased 5-hydroxymethylcytosine, and high proliferative activity. Oncotarget.

[36] Xuan F., Liu N., Zhang B.X., Wen W.X., Wang Y.C., Zhang H.F., Wu X.L. (2025). High expression and regulatory mechanisms of ANGPT1 and HOXA3 in acute myeloid leukemia. Bull. Cancer.

[37] Yuan S., Xu N., Yang J., Yuan B. (2025). Emerging role of PES1 in disease: A promising therapeutic target?. Gene.

[38] Zazuli Z., Irham L.M., Adikusuma W., Sari N.M. (2022). Identification of Potential Treatments for Acute Lymphoblastic Leukemia through Integrated Genomic Network Analysis. Pharmaceuticals.

[39] Joshi K., Zhang L., Breslin S.J.P., Kini A.R., Zhang J. (2022). Role of TET dioxygenases in the regulation of both normal and pathological hematopoiesis. J. Exp. Clin. Cancer Res..

[40] Bahadori F., Demiray M. (2017). A Realistic View on “The Essential Medicinal Chemistry of Curcumin”. ACS Med. Chem. Lett..

[41] Guo X.J., Huang X.Y., Yang X., Lu J.C., Wei C.Y., Gao C., Pei Y.Z., Chen Y., Sun Q.M., Cai J.B. (2023). Loss of 5-hydroxymethylcytosine induces chemotherapy resistance in hepatocellular carcinoma via the 5-hmC/PCAF/AKT axis. Cell Death Dis..

[42] Liang G., Wang L., You Q., Cahill K., Chen C., Zhang W., Fulton N., Stock W., Odenike O., He C. (2023). Cellular Composition and 5hmC Signature Predict the Treatment Response of AML Patients to Azacitidine Combined with Chemotherapy. Adv. Sci..

[43] Gong D., Zhang Q., Chen L.Y., Yu X.H., Wang G., Zou J., Zheng X.L., Zhang D.W., Yin W.D., Tang C.K. (2019). Coiled-coil domain-containing 80 accelerates atherosclerosis development through decreasing lipoprotein lipase expression via ERK1/2 phosphorylation and TET2 expression. Eur. J. Pharmacol..

[44] Cao D., Jia Z., Wu Y., Su T., Zhao D., Wu M., Tsukamoto T., Oshima M., Jiang J., Cao X. (2020). Demethylation of the RB1 promoter concomitant with reactivation of TET2 and TET3 impairs gastric carcinogenesis in K19-Wnt1/C2mE transgenic mice. Life Sci..

[45] Yoon M.J., Kang Y.J., Lee J.A., Kim I.Y., Kim M.A., Lee Y.S., Park J.H., Lee B.Y., Kim I.A., Kim H.S. (2014). Stronger proteasomal inhibition and higher CHOP induction are responsible for more effective induction of paraptosis by dimethoxycurcumin than curcumin. Cell Death Dis..

[46] Lindblad O., Li T., Su X., Sun J., Kabir N.N., Levander F., Zhao H., Lu G., Ronnstrand L., Kazi J.U. (2015). BEX1 acts as a tumor suppressor in acute myeloid leukemia. Oncotarget.

[47] Semizarov D., Glesne D., Laouar A., Schiebel K., Huberman E. (1998). A lineage-specific protein kinase crucial for myeloid maturation. Proc. Natl. Acad. Sci. USA.

[48] Kucuk C., Iqbal J., Hu X., Gaulard P., De Leval L., Srivastava G., Au W.Y., McKeithan T.W., Chan W.C. (2011). PRDM1 is a tumor suppressor gene in natural killer cell malignancies. Proc. Natl. Acad. Sci. USA.

[49] Li L., Ying J., Li H., Zhang Y., Shu X., Fan Y., Tan J., Cao Y., Tsao S.W., Srivastava G. (2012). The human cadherin 11 is a pro-apoptotic tumor suppressor modulating cell stemness through Wnt/beta-catenin signaling and silenced in common carcinomas. Oncogene.

[50] Meissner A., Gnirke A., Bell G.W., Ramsahoye B., Lander E.S., Jaenisch R. (2005). Reduced representation bisulfite sequencing for comparative high-resolution DNA methylation analysis. Nucleic Acids Res..

[51] NuGen Technologies (2023). trimRRBSdiversityAdaptCustomers.py [Computer software]. GitHub. https://github.com/nugentechnologies/NuMetRRBS/blob/master/trimRRBSdiversityAdaptCustomers.py.

