# Supplementary material for: Curcuminoids Activate TET Enzymes and Increase DNA Hydroxymethylation and Active Demethylation in Leukemia Cells
Source: Int J Mol Sci. 2025 Dec 27;27(1):310. doi: 10.3390/ijms27010310 (PMC12785487; doi:10.3390/ijms27010310)
Supplement: Supplementary file 1 [file ijms-27-00310-s001.zip › ijms-4049661-supplementary.pdf]

# Supplementary Data

## Supplementary Figure S1

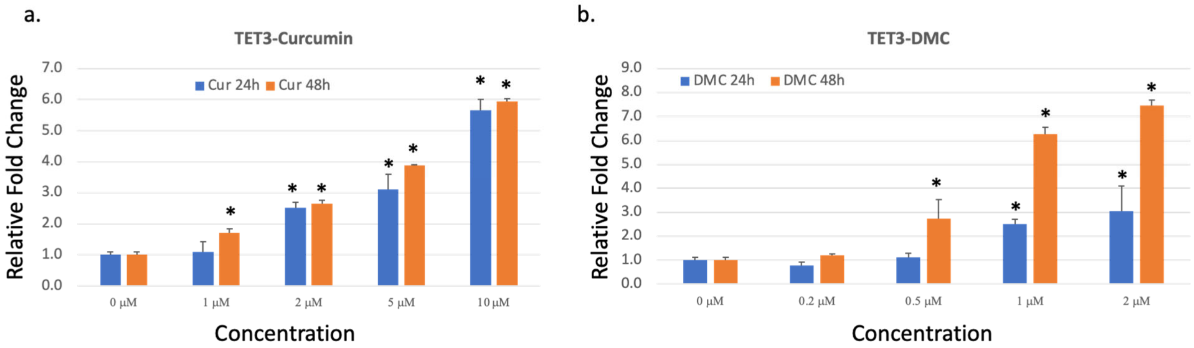


**Supplementary Figure S1. Curcuminoids induce TET3 isoform transcription in leukemia cells.** U937 leukemia cells were treated with graded concentrations of either curcumin or DMC (3a and 3b, respectively) for 24 and 48 hours followed by RNA extraction and single step RT-PCR as described under methods. The data represent the mean ± SD for 3 replicates. * indicates significant difference at $p<0.05$.

**Supplementary Figure S2**

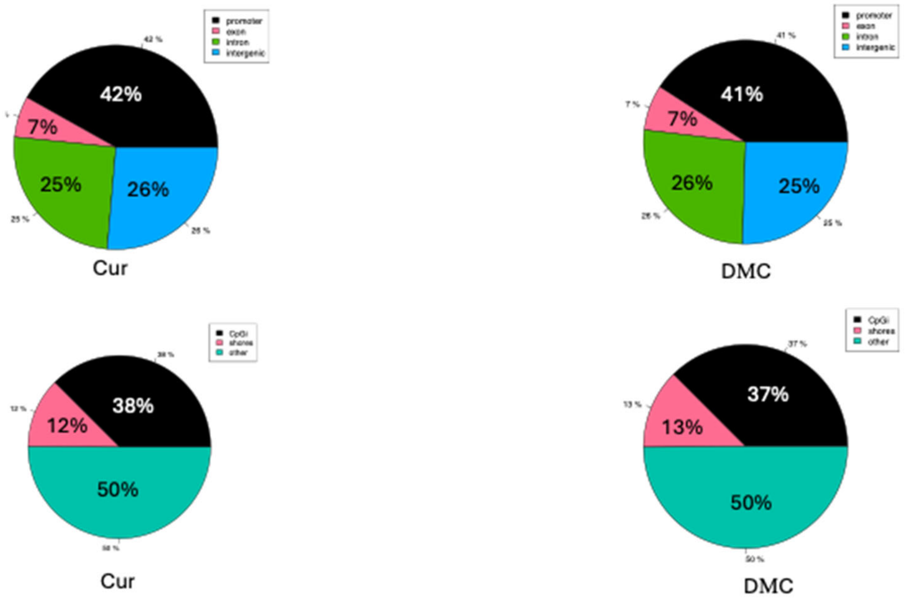


**Supplementary Figure S2. Genomic distribution of the decrease in 5hmC (active demethylation) induced by curcumin and DMC in leukemia cells.** U937 leukemia cells treated by curcumin (Cur) (5 uM) or DMC (1uM) for 48 hours followed by single CpG analysis of 5hmC distribution using RRBS as described under methods. The upper panel shows the distribution of 5hmC decrease in promoters, exons, introns and intergenic regions after treatment with Cur or DMC. The lower panel shows the distribution of 5hmC decrease in CpG islands (CpGi), shores and other regions after treatment with Cur or DMC.

**Supplementary Table S1. Leukemia-related genes showed 5hmC increase in promoter regions after DMC treatment.** U937 leukemia cells treated with DMC (1μM) for 48 h followed by genome-wide analysis of 5hmC as described under methods. Only genes with q-value less than or equal 0.05 were considered significantly different from the control.

| GENENAME | SYMBOL | q-Value |
|---|---|---|
| armadillo repeat containing 7 | ARMC7 | 0.0000 |
| brain expressed X-linked 1 | BEX1 | 0.0000 |
| CDP-diacylglycerol synthase 1 | CDS1 | 0.0000 |
| cytochrome P450 family 2 subfamily C member 19 | CYP2C19 | 0.0052 |
| FGR proto-oncogene, Src family tyrosine kinase | FGR | 0.0000 |
| interleukin 23 subunit alpha | IL23A | 0.0391 |
| kinesin family member C3 | KIFC3 | 0.0000 |
| long intergenic non-protein coding RNA 445 | LINC00445 | 0.0001 |
| long intergenic non-protein coding RNA 968 | LINC00968 | 0.0000 |
| microRNA 1972-1 | MIR1972-1 | 0.0001 |
| microRNA 4287 | MIR4287 | 0.0345 |
| microRNA 585 | MIR585 | 0.0291 |
| MNAT1 component of CDK activating kinase | MNAT1 | 0.0000 |
| MORC family CW-type zinc finger 1 | MORC1 | 0.0000 |
| myelin transcription factor 1 | MYT1 | 0.0032 |
| NLR family pyrin domain containing 1 | NLRP1 | 0.0257 |
| neurotrophin 3 | NTF3 | 0.0000 |
| opioid receptor mu 1 | OPRM1 | 0.0220 |
| pyruvate dehydrogenase kinase 2 | PDK2 | 0.0001 |
| protein phosphatase 2 regulatory subunit B'gamma | PPP2R5C | 0.0000 |
| pregnancy specific beta-1-glycoprotein 6 | PSG6 | 0.0001 |
| pituitary tumor-transforming 3, pseudogene | PTTG3P | 0.0064 |
| RAS p21 protein activator 4D, pseudogene | RASA4DP | 0.0000 |
| RAR related orphan receptor C | RORC | 0.0004 |
| sarcosine dehydrogenase | SARDH | 0.0000 |
| semaphorin 4A | SEMA4A | 0.0056 |
| SERTA domain containing 4 | SERTAD4 | 0.0125 |
| solute carrier family 2 member 9 | SLC2A9 | 0.0000 |
| small integral membrane protein 30 | SMIM30 | 0.0000 |
| small nucleolar RNA, C/D box 116-21 | SNORD116-21 | 0.0000 |
| ssu-2 homolog | SSUH2 | 0.0005 |
| SYNPR antisense RNA 1 | SYNPR-AS1 | 0.0001 |
| transcription elongation factor A like 5 | TCEAL5 | 0.0052 |
| ubiquitin conjugating enzyme E2 V1 | UBE2V1 | 0.0000 |
| WD repeat domain 64 | WDR64 | 0.0003 |
| zinc finger protein 415 | ZNF415 | 0.0003 |

**Supplementary Table S2. Leukemia-related genes showed 5hmC increase in promoter regions after curcumin treatment.** U937 leukemia cells treated with curcumin (5 μM) for 48 h followed by genome-wide analysis of 5hmC as described under methods. Only genes with q-value less than or equal 0.05 were considered significantly different from the control.

| GENENAME | SYMBOL | q-value |
|---|---|---|
| ankyrin repeat domain 20 family member A1 | ANKRD20A1 | 0.0000 |
| asialoglycoprotein receptor 2 | ASGR2 | 0.0000 |
| acyl-CoA wax alcohol acyltransferase 2 | AWAT2 | 0.0000 |
| caspase 8 | CASP8 | 0.0080 |
| C-type lectin domain family 12 member A | CLEC12A | 0.0000 |
| cytochrome P450 family 2 subfamily C member 8 | CYP2C8 | 0.0000 |
| cytochrome P450 family 4 subfamily F member 11 | CYP4F11 | 0.0000 |
| DLC1 Rho GTPase activating protein | DLC1 | 0.0035 |
| DnaJ heat shock protein family (Hsp40) member B3 | DNAJB3 | 0.0092 |
| dual specificity phosphatase 5 pseudogene 1 | DUSP5P1 | 0.0198 |
| GAS5 antisense RNA 1 | GAS5-AS1 | 0.0008 |
| glutathione S-transferase mu 2 | GSTM2 | 0.0031 |
| histidine decarboxylase | HDC | 0.0018 |
| immediate early response 5 like | IER5L | 0.0000 |
| keratin 16 pseudogene 2 | KRT16P2 | 0.0000 |
| leukocyte immunoglobulin like receptor A1 | LILRA1 | 0.0000 |
| long intergenic non-protein coding RNA 114 | LINC00114 | 0.0316 |
| long intergenic non-protein coding RNA 460 | LINC00460 | 0.0329 |
| muscleblind like splicing regulator 2 | MBNL2 | 0.0041 |
| microRNA 4436a | MIR4436A | 0.0000 |
| microRNA 4507 | MIR4507 | 0.0000 |
| microRNA 876 | MIR876 | 0.0000 |
| MT-RNR2 like 10 (pseudogene) | MTRNR2L10 | 0.0000 |
| neuroblastoma associated transcript 1 | NBAT1 | 0.0000 |
| NLR family pyrin domain containing 12 | NLRP12 | 0.0001 |
| protocadherin beta 10 | PCDHB10 | 0.0000 |
| protein tyrosine phosphatase receptor type D | PTPRD | 0.0000 |
| RNA, 5S ribosomal 1 | RNA5S1 | 0.0000 |
| secreted frizzled related protein 4 | SFRP4 | 0.0000 |
| solute carrier family 11 member 1 | SLC11A1 | 0.0446 |
| small nucleolar RNA, C/D box 116-4 | SNORD116-4 | 0.0421 |
| transmembrane protein 45A | TMEM45A | 0.0000 |
| VOPP1 WW domain binding protein | VOPP1 | 0.0000 |
| zinc finger and SCAN domain containing 5C | ZSCAN5C | 0.0000 |

**Supplementary Figure S3**

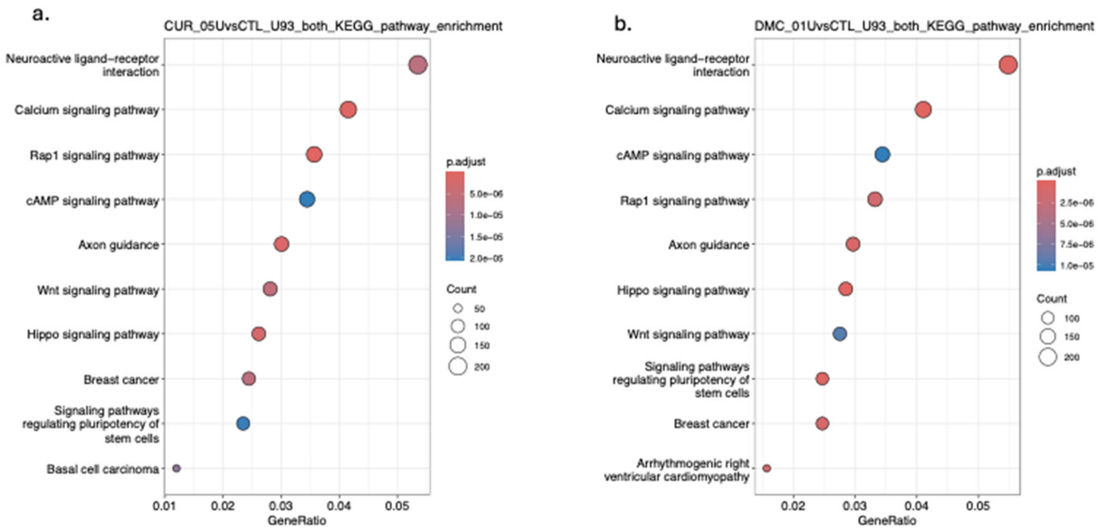


**Supplementary Figure S3. KEGG pathway enrichment analysis of 5hmC changes induced by curcumin and DMC.** U937 leukemia cells treated with either 5 uM curcumin (a) or 1 uM DMC (b) for 48 hours followed by oxidative bisulfite sequencing and genome-wide analysis of 5hmC mark as described under methods. Gene Ratio is defined as the number of the genes in the differentially hydroxymethylated sites, belonging to the specific gene ontology (GO) term to the number of all genes associated with differentially hydroxymethylated sites. For example, Wnt signaling pathway as a GO term with a gene ratio of approximately 0.03, means that out of every 100 genes that showed differential hydroxymethylation after drug treatment, 3 genes were involved in the Wnt signaling pathway. P. adjust indicates the adjusted p values and the size of the circles is proportional to the number of genes as shown in the figure key.
